# Beyond the Gender of the Livestock Holder: Learnings from Intersectional Analyses of PPR Vaccine Value Chains in Nepal, Senegal, and Uganda

**DOI:** 10.3390/ani12030241

**Published:** 2022-01-20

**Authors:** Renata Serra, Nargiza Ludgate, Katherine Fiorillo Dowhaniuk, Sarah L. McKune, Sandra Russo

**Affiliations:** 1 Center for African Studies, University of Florida, Gainesville, FL 32611, USA; smckune@ufl.edu; 2 International Center, University of Florida, Gainesville, FL 32611, USA; nrakhimova@ufic.ufl.edu (N.L.); srusso@ufic.ufl.edu (S.R.); 3 Master of Sustainable Development Practice, Gainesville, FL 32611, USA; k.fiorillo@ufl.edu; 4 Department of Environmental and Global Health, University of Florida, Gainesville, FL 32610, USA

**Keywords:** smallholder livestock systems, vaccine value chain, *peste des petits ruminants* (PPR), community animal health worker (CAHW), gender, intersectionality, Nepal, Senegal, Uganda

## Abstract

**Simple Summary:**

The *peste des petits ruminants* (PPR) is one of the deadliest viral diseases of small ruminants and is endemic in Africa, the Middle East, and Asia. While PPR vaccination programs exist in these contexts, many factors restrict their reach and effectiveness, which go beyond the often cited financial, technological, and logistical constraints. This paper examines these less investigated factors, in particular the role of gender norms and other social and cultural factors, in affecting which livestock keepers are unable to access PPR vaccines and which groups are less reached by the vaccine distribution systems. Across three countries selected for our study, we find that, overall, women derive fewer benefits from the system than men; in addition, belonging to certain ethnic groups, being of low caste, or living in remote regions are additional markers of exclusion and marginalization from accessing livestock vaccines.

**Abstract:**

The *peste des petits ruminants* (PPR) is a deadly viral disease of small ruminants, which are an important source of livelihood for hundreds of millions of poor smallholders throughout Africa, the Middle East, and Asia. PPR vaccination efforts often focus on overcoming financial, technological, and logistical constraints that limit their reach and effectiveness. This study posits that it is equally important to pay attention to the role of gender and other intersecting social and cultural factors in determining individual and groups’ ability to access PPR vaccines or successfully operate within the vaccine distribution system. We compare three study contexts in Nepal, Senegal, and Uganda. Qualitative data were collected through a total of 99 focus group discussions with men and women livestock keepers and animal health workers, 83 individual interviews, and 74 key informant interviews. Our findings show that there are not only important gender differences, but also interrelated structures of inequalities, which create additional sites of exclusion. However, these intersections are not generalizable across contexts—except for the intersection of gender and geographic remoteness, which is salient across vaccine distribution systems in the three countries—and social markers such as caste, ethnicity, and livelihood are associated with vulnerability only in specific settings. In order to address the distinct needs of livestock keepers in given settings, we argue that an intersectional analysis combined with context-dependent vaccination approaches are critical to achieving higher vaccination rates and, ultimately, PPR disease eradication by 2030.

## 1. Introduction

Gender analyses of livestock value chains have contributed to advancing our understanding of gender roles and dynamics across a variety of livestock production systems in low- and middle-income settings [1]. Such studies find that women’s contributions, while crucial, are hidden and given low social recognition, while men are predominant actors in the most lucrative activities and nodes, where profits and social connections usually abound [2]. Interventions that make women more visible, equip them with the right type of training and inputs, and foster open discussions about gender roles at the household and community level, have the potential to improve women’s gains as well as raise the economic and social performance of livestock systems [3]. The application of gender-sensitive tools and approaches to livestock research and programming has, however, been very uneven. In particular, there is a dearth of gender analyses pertaining to livestock health in low- and middle-income countries, despite the recognition that gender and other social factors affect livestock health as well as the performance of veterinary interventions [4,5,6]. This paper contributes to filling this gap by conducting an analysis of gender and intersectional issues within the system that provides veterinary services for small ruminants, with particular focus on the administration of the vaccine against the peste des petits ruminants (PPR).

PPR is one of the deadliest viral livestock diseases globally. During outbreaks, it can spread rapidly among herds of sheep and goats and kill up to 90% of infected animals. PPR is common throughout Africa, the Middle East and Asia, where small ruminants are an important source of livelihood for hundreds of millions of poor smallholder farmer households. PPR outbreaks each year create huge economic and social losses, in terms of reduced household income, greater food insecurity and diminished country export potential. Launched by the Food and Agriculture Organization (FAO) and the World Organization for Animal Health (OIE), the PPR Global Control and Eradication Strategy (PPR GCES) entails three key objectives, which are: to control and eradicate PPR by 2030, to reinforce Veterinary Services, and to improve animal health globally by reducing the impact of other major infectious diseases of small ruminants [7]. These three objectives require countries to improve and make progress along five technical elements: PPR diagnostic systems, PPR surveillance systems, PPR prevention and control systems, legal frameworks for PPR prevention and control, and stakeholder involvement [8]. However, PPR eradication and control through vaccination remain constrained in low- and middle-income countries by limitations in financial, human, and institutional capacities.

Vaccination coverage and effectiveness do not depend only on technical and logistical constraints. Rather, key social and cultural factors, preferences and norms—especially those related to gender—govern livestock keepers’ access to and use of vaccines [9]. This is because smallholder livestock systems—which include the majority of livestock keepers in Africa and Asia—are highly gendered spaces, whereby the distribution of roles and responsibilities of men and women follow quite rigid social and cultural norms. In most contexts, women do not formally own small ruminants, even while they care for them, and do not have access to the money, transportation, or a husband’s permission to call a veterinarian, take animals to vaccination sites, or receive veterinary services [1,10]. 

Previous studies have acknowledged the inequities coming from this rigid division of roles and argued for the need for veterinary services to address constraints due to existing gender norms if they are to reach the many animals cared by women [11,12]. We argue that veterinary services must go even further, by confronting other constraints beyond the sex of livestock holders. Not all men and women are the same: other aspects of their identity, such as age, caste, ethnicity, education, and geographic location, affect the extent to which livestock keepers and actors in the veterinary distribution system are able to access and, respectively, administer animal health care and vaccines. Few studies have identified the role of other social markers in affecting livestock vaccine uptake among small holders, for instance caste [13]. This paper contributes to an emergent field of study that analyzes how gender and other intersectional factors simultaneously influence smallholder engagements in the PPR vaccination systems. By uncovering hitherto unexplored constraints to countries’ PPR vaccination efforts, this research ultimately helps pinpoint opportunities that can be leveraged to make these systems more effective at reaching the marginalized and excluded. While intersectionality has a history of application in the legal field and public health research programming and is now increasingly deployed as an analytical lens within the feminist literature to examine power dynamics and structures of inequality, it has so far made limited inroads into research on agricultural and livestock production systems—let alone in research focused on veterinary services. 

We compare three study contexts in Nepal, Senegal, and Uganda. These contexts differ significantly in terms of geographic, technological, and socio-cultural characteristics, but they have in common that small ruminants play a key role in rural livelihoods, especially for women, and that PPR is a major challenge, which current interventions have not managed to prevent or control. By layering the use of intersectionality with a comparative study approach, whereby data have been collected following similar methodologies, this study is well equipped to analyze the complexity of relationships among multiple social groups [14]. The overarching goal is to pinpoint which intersections between gender and other social characteristics are common across vaccine distribution systems in the three sites, and thus more likely to be generalizable to other settings; and which social markers are instead associated with vulnerability only in a specific setting, and thus more context-dependent. 

## 2. Materials and Methods

### 2.1. Study Sites

**Figure 1:**
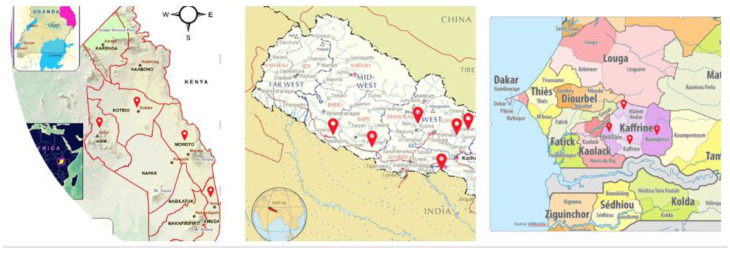
Study sites in Uganda, Nepal and Senegal.

The study was conducted in three countries (Nepal, Senegal, and Uganda), which reflect the varied ecological, economic, and social conditions of the two continents where PPR occurs; Asia and Africa (Figure 1). Study areas within the three countries were selected based on the importance of livestock keeping as a key livelihood source, the prevalence of PPR disease, the existence of notable gender and social dynamics, and the opportunity the sites presented to leverage experience and learning from previous research projects. In Nepal, four provinces were selected: Bagmati and Gandaki in the Hills agro-ecological zone, and Province 2 and Lumbini in the Terai agro-ecological zone. Mixed crop-livestock systems with goat rearing are predominant and valuable livelihood strategies to smallholders in these areas. These efforts are negatively affected by factors such as disease outbreaks, geographic isolation, limited access to markets and transport, and conservative Hindu caste-based social dynamics. Poorer households tend to be lower caste (Dalit) and reside farther from community and other resources available to higher caste groups (Brahmin and Chettri). In Senegal, the study took place in the Kaffrine region, located southeast of Dakar and in the center of the country. Many households in the region practice a mixed livestock–crop system of farming, including cash crops and small ruminants. When moving towards the northern and eastern parts of Kaffrine region, which are more arid and geographically more isolated, populations are more mobile and less dependent on crop farming and have increasing numbers of small and large ruminants. The ethnic mix changes as well, with Wolof groups more predominant in the crop farming areas and Fulani relatively more common in the northern and eastern portions where herding of cattle and small ruminants dominate the livelihood landscape. In Uganda, the Karamoja Sub-region, located in the northeast part of the country, was selected as a research site (specifically the four districts of Abim, Amudat, Kotido and Moroto). The region is home to the Karamojong ethnic groups such as the Jie, Matheniko, and the Ethur, and several other distinct ethnic groups such as the Tepeth and the Pokot. Agro-pastoralism represents the main source of livelihood for these populations, with many practicing pastoralism. These groups are geographically isolated and have been historically marginalized, socially and politically. The border with Kenya is highly porous with mobile pastoralists from both sides routinely crossing the border, in search of grazing lands for their herds, livestock markets, and services. Heavily armed cattle raiding of large herds has reemerged in the past two years, exacerbating inter-ethnic and gender-based violence.

### 2.2. Our Approach

A gendered intersectional analysis was applied to the PPR vaccine distribution systems in Nepal, Senegal and Uganda. This allowed for comparison of findings and to learn from the commonalities and differences in those systems. 

Gendered intersectional analyses investigate beyond dichotomic gender differences (that is, male/female) to explore how different groups of men and/or women experience given opportunities or constraints, and which sub-groups are more likely to be advantaged or disadvantaged. First introduced by Crenshaw, 1989 [15], intersectionality shows how multiple and interrelated structures of inequalities (gender, race, ethnicity, age, and class) can have multiplier effects when they intersect for a particular individual or group. As Collins, 2015 [16] puts it, an intersectional analysis is “an analytical strategy” that aids in a better understanding of how complex power relations result from intersecting gender with social status, ethnicity, caste, or other social markers. To be clear, intersectionality analysis is not about identity, per se, but about how identities make a difference in terms of understanding the structures of power, inequality, and (dis)advantage. Recent intersectional analyses of COVID-19 impact, for instance, show the insufficiency of pointing to a simple gender divide to highlight women’s burden from the COVID-19 pandemic. While indeed women in the US were more likely than men to take care of children and other family members during lockdowns, it was women of color and those from other minority groups who bore the greatest burden from the pandemic, in terms of job insecurity, exposure to the disease and associated health, material and psychological effects [17,18]. 

We utilize the concept of PPR vaccine value chain (VVC) to refer to the system of actors, knowledge and functions that characterize the supply and distribution of the PPR vaccine and associated services, from the central level to the end user. The PPR VVC in each study site was mapped and analyzed in other publications (see for instance, for Senegal, McKune et al., 2021 [19]) and is not the main object of this paper. Unlike the concept of supply chains, which focuses on the logistics of goods or service provision, the concept of the value chain is wider, as it encompasses not only material flows between actors, but also the information, knowledge and social capital that are shared between them [20]. Furthermore, value chains include not just formal but also informal aspects, which happen to be a more relevant component of the veterinary systems in low- and middle-income countries. The main actors in the PPR VVC, from the highest node down to the lowest level are: international or national vaccine producers, importers (if applicable), the veterinary drug and vaccine regulatory body, the responsible Ministry (e.g., Livestock or Agriculture), veterinarians and other specialized personnel at regional level, the animal health centers and personnel at provincial or district levels (e.g., veterinarians, livestock technical agents, or other specialized paravet personnel), community animal health workers (CAHWs) both formally or informally trained and dispersed throughout the territory and, finally, livestock keepers and their households. 

The combined gendered intersectional analysis is used to uncover overlapping sites of exclusion and inequity among these diverse actors from national, to regional/provincial, district, and community level. Qualitative data are disaggregated by gender and other social markers, identified according to prior hypotheses about which ones may be the most relevant in each specific setting. We selected gender and caste in Nepal, gender and ethnicity in Uganda, and gender and livelihood type (proxied by ethnicity) in Senegal. Our previous experience of working in these settings as well as secondary literature indicated that these intersections are embedded in the social fabrics of the target population and are among the most significant contributors to inequalities and power imbalances. We hypothesize that the vaccine value chains in all three contexts are both significantly gendered and highly intersectional, and that such intersections are key to explaining which type of women livestock keepers and women veterinary personnel can access, and, respectively, administer, PPR vaccines and related animal care. 

### 2.3. Methods of Data Collection and Data Descriptions

Qualitative methods were used to collect data across the three countries. These included literature review, Focus Group Discussions (FGDs), Key Informant Interviews (KIIs), and semi-structured individual interviews (IIs). Data were collected between July 2019 and February 2020 by mixed gender teams that included local research assistants as well as international graduate students, under the guidance of the principal investigators and country coordinators. All instruments and methods were subject to Institutional Review Board (IRB) approval by the University of Florida and associated country research clearance, where necessary (Uganda). Interviews and focus groups were conducted in national and local languages, and followed the same broad guidance, with some adaptations as appropriate to each local context.

Separate FGDs were conducted with livestock keepers and CAHWs, as well as with men and women within those categories—unless specified otherwise. The FGDs with livestock keepers focused on questions regarding local knowledge of and experience with raising small ruminants, gender roles, access to animal health services and vaccines, and a discussion of the advantages, disadvantages, and barrier to vaccine access (see Supplementary File S1 for the list of questions for this and all other instruments). The FGDs with CAHWs investigated the day-to-day responsibilities of local service providers and their experience working with communities. A total of 99 FGDs took place across the three countries. The FGDs with livestock keepers were disaggregated by gender, and where necessary, also by other social markers (by caste in Nepal, and ethnicity in Uganda) to allow for women and other vulnerable populations to speak freely. Questions were translated into local languages as needed. In locations where there were too few women animal health workers, the FGDs with CAHWs were mixed genders.

The scope of KIIs was to hear from experts from the public and private sector, government officials, and international actors at different levels of the VVC. Some interviews also included key stakeholders at the community level (e.g., community leaders, women’s group leaders, cooperative chairs, etc.). Participants were identified using the snowball methodology, whereby participants were asked to identify additional individuals for interview. KIIs followed a semi-structured format: starting from a set of guiding questions (which can be found in the list of instruments reported in the Supplementary File S1) conversations veered into adjacent topics, according to the responses obtained. For this paper, we retained content from the interviews that made reference to the role and positionality of women and other vulnerable groups in the various nodes of the VVC, and the related attitudes and perceptions about this involvement. A total of 74 KIIs were conducted across the three countries. 

The IIs were conducted with livestock keepers and animal health workers and the aim was to understand respondents’ roles in the livestock systems and assess their decision-making power, specifically related to livestock vaccines. Questions were asked about respondents’ characteristics and intersectionality factors that could be associated with (barriers to) participation in the VVC. A total of 83 individual interviews were conducted with livestock keepers and animal health workers. A summary can be seen in Table 1. 

All data collected through the above-described instruments are qualitative in nature. Responses were captured through audio recording—which were then transcribed and translated into English (in the case of FGDs in Nepal and Uganda) and French (FGDs in Senegal)—or note writing on paper (KIIs and IIs). The latter were written, whenever possible, by a second member of the team, while the first person was posing the questions and interacting with respondents. Hand-written responses were then typed into a work document. All responses were analyzed through thematic analysis, which involved, first, identifying emerging themes, e.g., themes which are sufficiently referred to in responses to be considered as significantly characterizing the aspects under investigation; and second, coding of responses according to the themes, to be able to provide content to the themes. Six themes were retained for analysis in this paper (see Table 2), some relevant across all three study sites, while few are context specific. 

## 3. Results

Findings are described below by each of the themes that emerged as relevant and meaningful across respondents, and by country. Qualitative analysis does not lend itself to quantification nor does it count the frequency of responses. Rather, content or quotes from responses are provided to support the elaboration of each theme (these are referred to by indicating whether from FGD, KII or II, followed by W or M indicating, respectively, a woman’s or man’s response, and the location).

### 3.1. Gender Differences in Knowledge or Decision-Making

Holding FGDs with men and women livestock keepers separately allowed for a clearer understanding of gender differences regarding knowledge about animal diseases, understanding and concerns about vaccination and potential discrepancies in genders’ expectations about each other roles. 

In Nepal, gender differences among livestock keepers in terms of knowledge and understanding of vaccination were marginal in communities where women belonged to a goat cooperative. Both men and women were able to describe the general signs of the PPR disease and explain the benefits of vaccination. “The [PPR] disease caused diarrhea in my goat.” “Vaccine prevents the PPR disease.” “I learned about vaccine from a recent training.” “My goat with vaccine fetched good price in the market.” (FGDs W, M, Gairabari Chok, Madhi, RaptiSonari, Mohottari). In communities with no goat cooperatives or where joining a goat cooperative is not possible due to social marginalization (such as for most Dalits or small ethnic groups such as Chapang), neither men nor women knew about the PPR or vaccinations (FGDs M, W, Ratmate, Tupche). In communities where men are frequently absent due to seasonal or long-term labor migration, men knew less and relied on women’s knowledge about the goat diseases, including PPR and vaccinations (FGDs M, Khayeramara, Phek); in many cases women have become de facto heads of households when men are absent. Male community-based animal health providers see women as the ‘first responders’ when an animal is sick, and those who often control the earnings from the sale of goats (FGDs CAHWs, Janakput, Nepalgunj, Kaski). These duties are seen as displays of strength and care they have for their families. Some women livestock keepers also suggested that in households where women own livestock the relationships with husbands were more harmonious (FGDs W, Gairabari Chok, Mijuri, Rapri Sonari). However, gender relations between men and women are not the same everywhere and differ by ethnicity and caste; in some groups, such as Newar, Magar, and among the Dalit, gender norms appear to be more equitable than in other groups/higher castes, allowing women and men within the households to make joint decisions about livestock. “When we decide with our wife savings take place, no haphazard expenses because women are more disciplined” (FGDs M, Rautahat).

In Senegal, both men and women in FGDs showed similar knowledge and ability to discuss a variety of animal diseases and their signs—all using local terminology. However, women’s groups seemed to be more “in the dark” with respect to what treatment to choose: “There are difficulties, because if this is for an illness and you do not know which treatment to get, then there is a problem, because when you call for a veterinarian, he comes very late. We are in the dark…” (GFD W, Keur Ablaye). Women admitted they lacked agency to bring animals to be treated, either because the small ruminants they care for belong to their husbands and not to them: “my husband has small ruminants, but it is me who takes care of them from morning to evening; …when I see that one animal is not well, I call my husband…so that he can call the vet… As [the vet] is not here, I call my husband and he calls the vet” (FGD W, Niahene); or because bringing animals for vaccination is considered as men’s task even for women’s herds: “we own [livestock] but it is my children who take care of animals…when there is a problem, it is up to them to know what to do and what they have to purchase”, and another: “I do not go; it is my son who goes and treats while waiting for the veterinarian” (FGD W, Keur Ablaye). Most women’s FGDs were adamant in pointing out that women do most of the work with animals, and they would know what to do; men are needed to give permission (FGD W, Kathiote). In some cases, women had found ways to negotiate with their husbands’ greater indepence: “In a way, if you explain to your husband well, he is not going to oppose. The problem is when you do something behind his back, but when he is informed, there is not problem” (FGD W, Niahene). Among livestock keepers, the ability to cite the benefits of vaccination and the difference between prevention and treatment was limited generally, with not much differences between women’s and men’s groups. Participants, men or women, in FGDs in Birkelana, Kaffrine and Malem Hodar were not able to name a vaccine available locally that could be used to protect their animals against PPR. In some communities, both men and women exhibited distrust of vaccine efficacy, due to experiences with vaccine delays and doubt regarding proper refrigeration of the vaccine (FGD W, M, Lours Escale). Some local animal health providers (called relais locally, to designate their role as community agents at the interface between local administrations and community members) attributed vaccination hesitancy among specific communities to lack of knowledge (KII 11 M, Gniby)—thus suggesting a communication gap between vaccine providers and livestock keepers. There were also gender differences in terms of the type of constraints to animal health. Most men livestock keepers mentioned poor access to feed and water as top constraints to animal health, in addition to irregular or distant veterinary services. Women tended to mention more often distant or inaccessible veterinary care (e.g., FGD W, Ndib Korki), the need to ask for husband’s permission (FGD W, Nihaè, Kathiote) on top of expensive or unreliable veterinary care (FGD W, Gniby, Mabo). 

In Uganda’s Karamoja sub region, goat rearing, and ownership vary among women of different ethnicities. The Jie and the Pokot rear and own goats and sheep, while the Tepeth and the Ethur women are not even aware when animals stay within manyattas (the traditional home structures). Across all ethnic groups, women have limited knowledge and decision-making power in relation to animal health and vaccination: “They don’t tell us the names of those vaccines. That is because we are not involved and we are not allowed to go there [cattle crush or kraal where vaccinations take place]” (FGDs, W, Alerek, Kotido, Loroo). Generally, women indicated low understanding of what vaccines are for, and lacked clarity in distinguishing between treatment and prevention. The (mainly male) CAHWs and veterinarians typically do not provide women with information about vaccination campaigns: “The problem is not only are women uninterested and they are very unconcerned but also the men do not want women around and they don’t think women also have a role to play in animal vaccination” (FGD W, Alerek). Furthermore, women who have some knowledge expressed concerns about taking the livestock to vaccination for fear of their husbands’ disapproval and even violence: “If your husband refuses to take animals for vaccinations and threatens to divorce you if you do, he will even beat you” (FGD W, Morulem). Most FGDs with women referred to their lack of decision-making power as ultimate problem. Even women whose husbands have died are expected to consult male in-laws or elders in decisions regarding livestock (FGD W, Alerek). 

### 3.2. Perceived Gender Differences in Abilities

In all three study contexts, there are not just objective differences in gender roles, but also differences in perceptions about what each gender can do and contribute. Again, by discussing with groups of men and women separately, and interviewing men and women actors in the value chain separately, it is possible to obtain an insight into gender related beliefs and social norms. Communities from all three study sites shared the overarching belief that women are unable to restrain not only large ruminants but also small ruminants, due to lack of physical strength as well as stamina. 

In Nepal, male CAHWs and junior technicians referred to women’s weakness when handling animals during various animal health check-ins, including vaccination, in the following terms: “It is difficult to handle animals. You need physical strength. Mostly women are there. Sometimes, we have to leave without treating animals, because women can’t hold animals” (FGD M CAHWs, Nepalgunj). Some men livestock keepers expressed lack of trust in women CAHWs, reputed to be weak physically or technically (FGD M, Binauna). Another explanation was centered around cultural beliefs. In marginalized communities (e.g., Madhesi and Chapang) there is the belief that women should not be allowed to touch animals (FDGs M, W, Baijapur, Binauna, Damar Bahanjyang).

In Senegal, most FGDs with men recognized the important roles that women played, but mostly as ancillary to the men (FGD M, Lours Escale); women’s lack of physical ability was often mentioned: “Women are great addition to livestock breeding/animal husbandry but when the heat comes the management of the livestock becomes difficult to handle for them” (FGD M, Kathiote). Several women’s FGDs, on the other hand, emphasized not only women’s essential contribution (FGD W, Gniby), but also the fact that women could handle more if they were given the chance to: “Women have received training and know how to vaccinate poultry… [They] would love to add small ruminants and vaccinate themselves. Anything men can do, women can do” (FG W, Touba); “Yes it would be possible [to vaccinate against PPR]; if I had a training, I could do it myself…everything that a man can do, a woman can do it too” (FGD W, Niahene). FGDs and interviews with livestock technical agents also referred, as an indisputable reality, to the fact that women lack the physical strength to restrain ruminants, large or small. Experts from livestock projects and veterinarians confirmed that women are trained and recruited mainly to vaccinate chickens and most relais are men (KII 9 F, KII 10 M). Furthermore, a male veterinarian noted that women livestock keepers, since they have fewer animals, have greater difficulty in access to vaccines “because they [only] need fewer doses and [unit] cost is comparatively large” (KII 4, Diamnadio).

In Uganda, women are largely perceived as too weak and ‘polite’ to restrain livestock during the vaccination process or fight through the crowds to take their animals to be vaccinated. Women are also perceived as being unable to care for healthy animals, despite the fact that most sick animals are left with women in manyattas, when men take healthy animals for grazing: “Women do not have the energy to even follow the livestock up to the mountains to drive them back to the cattle crash for vaccinations”; “If the vaccine is less and a woman comes first she can get. But if man comes first and a woman comes later she might miss because the women are polite in most cases, they are not able to [physycally] fight for vaccines” (FGDs, M, Alerek Karita). Another barrier is around women’s menstrual cycles, which prevent women from accessing livestock among the Pokot: “It is a bad omen for women who are bleeding to approach livestock” (FGD M, Karita, Amudat Town council). 

### 3.3. Gender Differences and Inequalities among Animal Health Providers

In Nepal, only 3 out of 12 veterinarians and 2 out of 11 paravets/CAHWs interviewed were women, which, considering active efforts to locate women for this study, reflects their minority status among veterinarians and paravet personnel. Gender-specific barriers discussed included: mountainous terrain and inferior infrastructure in rural areas that complicate the movement of women CAHWs from one location to another, early marriages that prevent young women from completing school, and household chores preventing women from exploring opportunities outside their homes. Low literacy and numeracy among women were also cited in KIIs, further limiting women’s ability to attend technical schools (KIIs, Kaski, Chitwan). Interviewed CAHWs cited the lack of trust among male livestock keepers when women animal health providers attend to the animals (Mixed genders FGDs, Kaski, Mahottari). An interesting aspect that emerged is the narrowing of gender inequalities among CAHWs as more women have receved training in the last decade to treat and vaccinate goats, thanks to NGOs and the government’s efforts to better serve women livestock keepers. The feminization of agriculture and livestock in Nepal, as well as remittances from abroad from men who have migrated help encourage more women to diversify their occupations and seek opportunities in veterinary services. Several women CAHWs said that they wanted to become CAHWs to move away from working in rice fields. The creation of women-led goat cooperatives was observed as another positive trend that is jolting the social norms that women are born to look after their husbands and raise children.

In Kaffrine, Senegal, during data collection, the regional livestock inspector was a woman, but all four district inspectors were men, as were 17 out of 21 livestock technical agents. Women comprise a minority of CAHWs according to informants’ accounts, although relais are mainly informal and there is no official record of numbes and sex. The few women in the system face greater constraints in their work when compared to men. According to the president of a women’s livestock association, widespread illiteracy among rural women is a serious obstacle, which discourages the training of women as relais (KII 7 W, Kaffrine). Women’s lack of motorbikes—due to social norms preventing women from riding motorbikes alone, but also women’s lack of financial means to buy and maintain such an expensive means of transport or to use it after dark—restricts their work and prevent them from reaching a wide number of livestock keepers, especially since communities in Kaffrine are scattered and some are very remote. Furthermore, even when women enter higher rungs in the veterinary system in Senegal, they tend to lag behind in terms of career prospects. This is due in part to lower educational achievements, but for the other part to the fact that, as wives and mothers, women enjoy a lower degree of mobility than their male counterparts: “No woman is currently chief of livestock post…this is a difficult job, requiring mobility, and not easy for women to do it” (KII 8, W). An informant from a livestock development project also shares that “it is difficult for women to go up the veterinary system, because they don’t have the education requisites” (KII 9, W). Since veterinary personnel in Senegal are required to transfer to another part of the country at regular intervals, lack of mobility on the part of women may limit their ability to rise to the top within the livestock system. Thus, gender inequalities remain pronounced, despite Senegal having made recent progress in reducing gender gaps in university enrolment and formal labor participation.

In Karamjoa, Uganda, in the four districts of the study, all 10 veterinarians were men, while only 3 of the 12 animal husbandy staff and assistants were women. The lack of women within the VVC is explained by multiple factors: reproductive household responsibilities, mobility constraints, non-functional gender policies, and rigid gender social norms. However, more importantly, women from other parts of Uganda are not interested in working in the harsh and often violence-prone Karamoja areas, while there is currently a lack of educated Karamojong women pursuing veterinary degrees. Moreover, current requirements to become a CAHW demand some level of literacy, whereas women in Karamoja have low literacy when compared to the national level. Additionally, the job of CAHWs is very hard: “What makes people hate this job is that if you handle an animal in a hard way, either they kill you or break your leg; your teeth may be knocked out as well” (CAHW FGD, M, Alerek). It is not very lucrative either even for men, as there is little compensation for the amount of work they are expected to perform: “…when they don’t even pay us we become a laughing stock of other people” (CAHW FGD, mixed genders, Kotido). Considering the amount of responsibilities women have already in the household, they are less inclined to seek work as a CAHW. 

### 3.4. Differences by Caste

As hypothesized, differences relating to caste in Nepal were found to be significant. The majority of actors and informants at the national, province, district, and rural municipality levels confirmed that lower castes are under-represented in the upper nodes of the vaccine systems and among veterinarians (KIIs, Kaski, Tanahu, Dhading, Nuwakot, Chitwan, Palpa, Kathmandu). For instance, 12 veterinarians interviewed through KII and 11 paraveterinarians interviewed through FGDs were from upper castes, Brahmin and Chettri. This was explained in part by unequal educational achievements between the castes, with low castes being more likely to be without formal education. Among livestock keepers, Dalit women face disproportionate constraints derived from the intersecting discrimination they experience due to gender and caste. Their animals are consequently less likely to be vaccinated or to benefit from animal health services. Dalit households are usually located at the fringes of villages, while vaccinations are held centrally, making it more challenging to take animals to vaccination sites. Lower caste households own fewer animals, thus diminishing any incentive for vaccinators to visit them. Dalit women, unlike higher caste women, often work for wages outside their home, and thus are more likely to be unavailable when vaccinators visit their villages: “It’s due to inappropriate time” (FGD W, Somarfulbari). Finally, Dalit women do not belong to goat cooperatives largely due to social stigma and unwillingness of higher caste women to accept Dalits as equal members of the cooperative: “Those Dalit women are not interested to participate [in a cooperative]” (FGDs, W, Khayeramara, Chandrapur, Shaktikhor).

### 3.5. Differences by Ethnicities

In Uganda, there are important ethnic differences that qualify and mediate gender differences [21]. Representation of ethnic groups from within the Karamoja sub-region is almost non-existent at higher VVC nodes. As described above, most professional veterinarians and livestock extension agents who work within the sub-region are from districts outside Karamoja or from Abim, and may not speak local languages [Abim populations speaks Leb Thur]. Among the Jie, women are more likely to be engaged in livestock activities than women from other ethnic groups. During FGDs, the Jie and the Pokot women exhibited higher understanding of the importance of vaccines (including the difference between prevention and treatment) and expressed a certain willingness to vaccinate. Furthermore, there are important variations by district, which reflect different gender traditions and norms of the ethnic groups. The Jie, Matheniko, and Tepeth women are more engaged in day-to-day livestock activities, while widows among the Jie and the Pokot can own livestock, and the Pokot allow women to sell animals at markets. Other groups (especially the Ethur, but also the Matheniko, Tepeth, and Pokot) have more restrictive cultural norms about women accessing livestock kraals, a traditional livestock enclosure in Karamoja, only allowing these interactions when the man is not available or when women are widows. Women’s access to livestock kraals is also restricted when they are menstruating: “There is a law that the young women who are having the sickness of the moon cannot enter the kraal” (CAHW FGD W, Alerek). 

In Senegal, FGDs hinted at some ethnic differences in the way gender roles affected women’s involvement with livestock. “Among the Wolofs, any woman who has the means can raise livestock, as nothing prevents her from doing so, but this is not the case among other ethnicities” (FGD W, Keur Ablaye). When Fulani women in the same FGD were asked what they thought about that, they confirmed: “It is as she said: when we have children, we stay at home”. However, Fulani women in other FGDs eagerly talked about their important roles in feeding and taking care of small ruminants, which are very important to their family livelihoods. 

### 3.6. Transhumance and Conflict

In Senegal, the type of livelihood significantly affects access to animal health services. The animal health workers interviewed confirmed that pastoralists are harder to reach and more likely to miss vaccination, since frequent delays in the vaccination campaigns imply that many herds have already departed (KII 13 M, Gniby). Livestock keepers “who live far away and don’t have young members to take animals to vaccination parks or live at the margins of villages, especially transhumant Fulani” are more likely to be missed (KII 12 W, Gniby). Some FGDs with men lamented the difficulty of moving herds to find sufficiently large corridors (FGD M, Touba-Mbella). In a few FGDs with men, pastoral populations were blamed for spreading animal diseases into their communities, especially when herds meet at water points, something that was echoed in women’s FGD: “There are animals who come from Walo and are already sick… When they come we share water points. Here, all herds from Walo, and Mauritania they mix” (FGD W, Malem Hodar). Women also referred to the frequent occurrences of robbery of herds as an increasing worrisome issue—provided sometimes as an explanation for their transitioning from small ruminants to poultry raising: “I used to raise sheep…but the last theft that I suffered…that is what made me start with poultry…with chickens there is no theft, because you build an enclosure and you close it” (FGD W, Malem Hodar). 

In Karamoja, Uganda, inter-ethnic conflict prevents some livestock owners from seeking vaccination: “Those ones [Matheniko and Turkana] are our enemies, they can’t vaccinate their animals here. They won’t even come to vaccinate their animals” (FGD W, Lokitelakaebu). Some livestock keepers also flagged vaccination campaigns as an opportunity for bad actors to identify animals to later raid and steal: “If the Matheniko came for vaccines, they will doubt that vaccines are the real intention, they think it would be to canvas the amount of animals they have to later steal them”. This practice of cattle raiding may also inhibit many people from bringing their animals to a central location: “These warriors come to see your animals and strategize, they don’t come for vaccinations” (FGD M, Morulem). Some livestock keepers indicated that Matheniko and Turkana can only access vaccines after peace has been established in the community (FGD M, Nakapelimoru). 

## 4. Discussion

Our analysis of data from three countries yields some common findings as well as results that are specific to each setting, thus underscoring the context-dependent nature of constraints to vaccine interventions. 

Two main findings speak to the highly gendered nature of PPR distribution systems in our three studied sites. The first is that, among livestock keepers, women assume many responsibilities for animal care, but their access to veterinary care is largely controlled by men in the household. Second, gender inequalities increase as one moves up the value chains, so that, while there is a general paucity of women among animal health providers at the community level, there are even fewer women who are veterinarians, heads of district or regional level offices. By intersecting gender with other social stratifications, our analysis identified additional characteristics of individuals and households that make them less visible to PPR vaccination services. 

The observed gender divide is stark, but perhaps not too surprising. It confirms findings from other studies, pointing to how women are relegated to lower nodes within livestock and agricultural value chains [22,23]. The differentiation between what are deemed women’s suitable spaces (poultry vaccination in Senegal) and men’s spaces (vaccination of ruminants large and small) echoes well known studies on the distinction between female and male crops [24,25]. As elsewhere, social norms underpinning such presumed gender differentiations tend to reinforce women’s economic and social disadvantages, since providing health care to small flocks of chickens is much less lucrative than providing care to larger animals. 

What is more surprising is that, despite the well-known gender differences and potential consequences, vaccine distribution interventions remain by and large gender neutral—and this can be said not just of the three studied contexts, but probably of most others as well (for the negative effects of gender-blind systems in similar contexts, see for instance Aregu et al., 2016 [26]). Gender neutral systems treat everyone the same. Gender-aware, and gender-transformative systems, instead, recognize that women and men face different constraints; they attempt to overcome, and, respectively, transform (the social norms that undergird) these constraints, in order to give everyone the same opportunity to benefit from a service. 

A third important finding from our study is that actors in these systems are aware of gender constraints. Indeed, many of our study participants know that vaccine services fail to reach the poorest and most vulnerable members within communities, and that women livestock keepers lack the mobility, autonomy, and decision-making power to take the best actions for their animals. Still, none of the existing policies or interventions explicitly address these constraints, nor do they go the extra mile to make up for such inequalities in access. A relevant question is why providers along the VVC do not take into explicit account existing disparities among livestock holders and thus continue to fall short of actively addressing the needs of the most vulnerable individuals and groups. We found that in Kaffrine, Senegal, this is partly due to a generalized sentiment that one’s community is poor—a blanket statement that fails to recognize the many social differences, along gender, economic, and social lines, that exist within communities [26]. In Karamoja, Uganda, where several distinct groups are present and many of the personnel originate from other parts of the country, limited cultural understandings of the context may be an obstacle to how personnel operate in the region. In Nepal, there are marked differences in social status and caste between personnel in the livestock VVCs, on the one hand, and livestock keepers who are being served, on the other hand; this contributes to reciprocal distrust, which negatively affects both the quality of service delivery and trust in the service provided.

The most innovative result from this study is that an improved understanding of the inequities of vulnerable groups requires going beyond gender and analyzing other social and spatial factors that create multiple overlapping sources of inequities; these intersecting factors play a critical role in designing effective vaccination programs. One overarching source of vulnerability, found to be common to all three contexts, is remote location. Distance from the main road, in regions characterized by poor infrastructures and lack of suitable transportation (and in the case of Nepal, mountainous terrain), invariably condemns remote livestock owners to lower access to services, other things being equal. More importantly, since remote and non-remote households often differ significantly in their other social characteristics, such as poverty status, educational attainment, ethnicity, and caste, geographic remoteness compounds rather than covaries the negative effects of these other sources of inequality on livestock production and engagement. The current incentive structures for livestock vaccination mean that vaccinators are least inclined to travel to and engage the populations who are most vulnerable to the negative outcomes of poor animal health. 

An improved understanding of how multiple social identities intersect to determine overlapping and compounding experiences of vulnerability is a necessary requirement, we argue, for designing vaccination efforts that avoid the limitations encountered in any given context. Below, we give some examples of how our findings could help modify existing interventions to improve their reach and inclusiveness strategies. 

In Nepal, inequities among castes partly override those determined by gender—as low caste women and men face disproportionate constraints to accessing quality education and resources and are unable to benefit from the VVC. In this context, a focused effort on reaching (women and men) livestock keepers from lower castes and most remote households would be a more effective strategy than aiming to vaccinate all women’s animals per se.

In Kaffrine, Senegal, instead, strong differences by gender, together with those by location, seem to be more marked than livelihood and ethnic differences. Patriarchal norms exclude women from either vaccinating small ruminants or deciding to have their animals vaccinated—and there is limited variation by ethnicity or social status. In Senegal, a greater attention to gender differences has been impeded by widespread prejudices against what are perceived to be alien or foreign concepts of gender—unfortunately creating confusion even among educated livestock agents and sometimes inhibiting genuine discussion, engagement, and action about gender issues in the local context. The need here is to create awareness around gender differentiated needs that may be locally acceptable. 

Remoteness, poverty, and underdevelopment combined with continued violence define the Karamoja sub-region even more than the other study sites. Consequently, vaccination interventions must overcome basic challenges of population dispersion, poor infrastructure, and lack of education and economic means. However, ethnicity and gender intersect to denote further areas of marginalization, as well as surprising areas of access. Because gender norms are more restrictive for some ethnic groups than others, veterinary services could improve their work by more nuanced targeting, such as deploying a more pro-active approach to gender sensitization in some contexts more than others. For example, the Ethur require more gender sensitive veterinary services to reach women and sensitize men on women’s role in animal health compared to the Jie and the Pokot. The Matheniko and the Tepeth need gender responsive interventions to expand women’s access to livestock ownership and resources. Expanding educational and career opportunities among Karamojong, especially women, would also reduce the need to employ veterinarians from other parts of the country. These strategies would allow the livestock vaccine systems to leverage ethnic and gender-specific knowledge for more effective veterinary services. 

## 5. Concluding Remarks

Gender-blind animal health interventions are incapable of addressing the gendered constraints embedded in livestock systems and will continue to fail to eradicate deadly livestock diseases, including PPR, in the affected countries. Gender-transformative interventions incorporate understanding of how gender differences intersect with other social markets to determine deeper sites of exclusion and attempt to overcome and change these. However, implementing an approach that only considers gender, and does not examine other factors such as remoteness, caste, ethnicity, and livelihoods, will fail to reach the most vulnerable populations. Consistently across the study areas, it was an intersection of several factors that illuminated different barriers to vaccine uptake and showed how complex those barriers might be. 

Vaccination campaigns must move beyond consideration of the gender of the livestock holder toward creation of policies and programs that foster equitable demand and access among different categories of livestock owners in order to achieve disease eradication. These changes, however, are hard to come by, since unequal gender and social norms are embedded in the social fabric and run throughout the technical services that provide services to the communities. We argue that, if animal health services are to implement a gendered intersectional approach, intensive training should be provided in gender issues, social inequalities, facilitation and conflict resolution. Such training should become a regular part of the technical education and sensitization of all actors within the system, from the top veterinarians to the rural communities, and its implementation should involve the public sector, NGOs and development community leaders and experts. In Senegal, for instance, such initiatives could work with gender unit within the Ministry of Livestock and Animal Production as well as with the National Center for Education of Technical Agents for Livestock and Animal Industries. While we do not want to detract from the importance of technical training, some of the constraints within the veterinary systems are a consequence of the personnel’s inability to address the differentiated needs of rural communities as well as communicate knowledge and information in culturally appropriate and accessible ways. By forming true extension agents who are aware of the complex issues affecting access to and demand for vaccines, veterinary systems will be better positioned to ensure that disease pockets do not persist during and after vaccination campaigns. 

## Figures and Tables

**Table 1:** Breakdown of qualitative instruments used in fieldwork, by gender and country *.

	Men	Women	Mixed Gender	Total
FGD-Nepal	13	13	4	30
FGD-Senegal	16	14	1	31
FGD-Uganda	18	16	4	38
**Total FGD Sessions**	**47**	**43**	**9**	**99**
KII–Nepal	24	6	*	30
KII–Senegal	17	6	*	23
KII–Uganda	17	3	1	21
**Total KIIs**	**58**	**15**	**1**	**74**
IIs–Nepal	15	15	*	30
IIs–Senegal	15	15	*	30
IIs–Uganda	12	11	*	23
**Total IIs**	**42**	**41**	*	**83**

* Instruments include: Focus Group Discussions (FGDs), Key Informant Interviews (KIIs) and Individuals Interviews (IIs). Totals are in bold.

**Table 2:** Themes identified in qualitative analysis, by country.

	Gender Gaps in Knowledge/Decision-Making	Perceived Differences in Abilities	Gender Differences, Animal Health Providers	Caste Inequalities	Ethnic Differences/Inequalities	Transhumance and Conflict
Nepal	X	X	X	X		
Senegal	X	X	X		X	X
Uganda	X	X	X		X	X

## Data Availability

Not Applicable.

## Supplementary Materials

The following supporting information can be downloaded at: https://www.mdpi.com/article/10.3390/ani12030241/s1, File S1: List of Instruments for Qualitative Data Collection.
